# A Novel Method for Calculation of Molecular Energies and Charge Distributions by Thermodynamic Formalization

**DOI:** 10.1038/s41598-019-56312-2

**Published:** 2019-12-30

**Authors:** TongIl Kim, ChungIl Ri, HakSung Yun, RyongNam An, GwangBok Han, SungIl Chae, GyongNam Kim, GwangChol Jong, Yung Jon

**Affiliations:** Institute of Chemistry and Biology, University of Science, Pyongyang, 950003 Democratic People’s Republic of Korea

**Keywords:** Chemistry, Physical chemistry, Thermodynamics

## Abstract

The paper describes a new approach to the thermodynamic formalization for calculation of molecular energy and charge distribution in ground state by means of the variational equation of DFT. In order to thermodynamically formalize the molecular calculation, the pseudo chemical potential (PCP) is conceptualized, where a molecule is broken into multi-phase(atom) one-component(electron) systems and the energy of system is represented as PCP. Calculation of the molecular energy and atomic charge by PCP is put forward, thereafter the approach is proved to be valid and its efficiency (accuracy and calculation speed) is verified.

## Introduction

In quantum theory for calculating molecular energy, atomic charge, structures and properties, excellent successes were achieved in calculation for many projects by establishment to ab initio and semi-empirical method of wave function theory (WFT)^1–6^ in that electron wave function is variable quantity of energy minimization and density-function theory (DFT)^7,8^ in that electron density is variable quantity.

Of course, the ab initio method of WFT is widely used for calculating the properties of the molecules because of its high accuracy^9–13^. However, it is infeasible for calculating the molecules with a large quantity of atoms, because the more quantity of atoms is given, the longer it takes to calculate. Therefore, less accurate DFT based on electron density has been created and used^14–20^. Over the past decades, much effort has been made to improve the calculation accuracy of DFT^7–15,21,22^. WFT, DFT and its varieties are all different in calculation accuracy and velocity, but both formalization of the variable quantity of energy minimization and the solution of the variational equation are based on the quantum theoretical formalization.

The aim of this paper is to lay foundations for the principles and methods which could determine the energy of molecules and their atomic charges by thermodynamic formalization, rather than the quantum theory.

In the variational equation which gives the electron density corresponding to minimum energy in DFT, Euler-Lagrange (EL) equation, an undefined multiplier μ is the energy per electron and electron migration measure.

1 $${\rm{\mu }}={(\frac{\partial E}{\partial N})}_{{v}({r})}$$

(μ: chemical potential, E: the energy of an electron system, N: number of electrons, $v(r)$: external potential).

This μ can be also defined in the ground state of a pure molecule equal to temperature zero limit state of finite temperature thermal equilibrium system. That is

2 $${\rm{\mu }}={(\frac{\partial A}{\partial N})}_{\theta ,v(r)}={(\frac{\partial E}{\partial N})}_{\theta ,v(r)}-\theta {(\frac{\partial S}{\partial N})}_{\theta ,v(r)}$$

3 $${\rm{\mu }}={(\frac{\partial E}{\partial N})}_{v(r)}=\mathop{\mathrm{lim}}\limits_{\theta \to 0}{(\frac{\partial A}{\partial N})}_{v(r)}$$

where *θ* is finite temperature of the thermal equilibrium system, A is the free energy of $\theta ,v(r)$ electron system and *S* is entropy of electron system under $\theta ,v(r)$.

A pure state molecule electron system, not a mixed one, can be supposed to be a thermodynamic system. In this regard, the state density and the state sum depend on the energy level and degeneracy in formalization of the universally used statistical models of electron system (statistical model^23^ of atom, statistical model^24^ of 2-atom molecule, gas model^25–30^ of free electron, Thomas-Fermi model^31,32^ of DFT), and from the statistical viewpoint, the molecule can be regarded as the thermodynamic system, based on the fact that the property of the macroscopic system, thermodynamic property, can be obtained by means of the state sum.

From such viewpoint, to applicate the method of thermodynamic formalization, we taken undefined multiplier μ in the EL equation as a function form proportional to the logarithm of the electron number and then defined it as the pseudo chemical potential (PCP). We divided the total energy, the quantum mechanical average value of molecule Hamilton operator, into atom sections and proposed a new molecule energy calculation model which is described as a PCP. Hence, the molecule energy is denoted by the function of electron number and electron number is made to become the variable quantity of energy minimization rather than wave function or electron density. The partial derivative value of the *α*-th atom’s electron number for the total molecular energy is defined and formalized as a pseudo chemical potential of each phase (atom) (PCPP).

4 $${\mu }_{\alpha }^{M}={(\frac{\partial {E}^{M}}{\partial {N}_{\alpha }})}_{v(r),{N}_{\beta \ne \alpha }}$$

On the basis of the above definition, we proposed the thermodynamic model of multi-phase one component molecular system, in which atoms are regarded as phase and electrons are regarded as component. In addition, variation principle is put forward, which means the molecule formation process is the minimization process of energy and equalizing process of each phase’s PCPP as well.

5 $${E}_{0}(N,v(r))={\inf }_{N}E(N)$$

The molecule is formed when PCPP of each phase is equally formed. From thermodynamic viewpoint, it is similar to the Gibbs’ phase equilibrium condition where the chemical potentials of each phase and component are equal. From the PCPP equilibrium condition, the simultaneous equation (variational equation) where the electron number *N* is an undefined number is obtained. The number of electrons, the solution of simultaneous equation, is a stationary value that gives the minimum value of energy in the ground state of the molecule and the value that determines the atom charge in the ground state as well. The thermodynamic formalization has been evaluated through the calculation experiment. What is unique in this method is that a new thermodynamic model for molecular electron system is proposed and calculating system for the molecular system is established by using the method of thermodynamic formalization. To the best of our knowledge, in previous researches, there has been no research that described the energies of the molecular electron systems as chemical potentials and the charge distribution of atoms in molecules is realized by the method of the thermodynamic formalization.

## Theoretical Foundation

### Definition of PCP by thermodynamic method

EL Eq. (3)^7,8,31^ of DFT

6 $${E}_{V}[\rho ]=T[\rho ]+{V}_{ne}[\rho ]+{V}_{ee}[\rho ]$$

7 $$\int \rho (r)dr=N$$

is obtained by variation of energy of electronic system (6) under constraint (7).

$$\delta \{{E}_{V}[\rho ]-\mu [\int \rho (r)dr-N]\}=0$$

8 $${\rm{\mu }}={[\frac{\delta E}{\delta \rho (r)}]}_{V}\,{\rm{or}}\,\mu ={[\frac{\partial E}{\partial N}]}_{V}$$

As mentioned above, for the sake of thermodynamic formulation, the undetermined multiplier *µ* is defined as the function in proportion to logarithm of electron number, hereafter called PCP, which is formulated as follows:

9 $$\mu ={\mu }_{0}+\gamma \,\mathrm{ln}\,\frac{N}{{N}_{0}}$$

where *N*_0_ is electron number of neutral atom, *N* is electron number of composed atom of molecule, µ_0_ is µ value when *N* = *N*_0_, γ is parameter.

10 $$E=\int \mu \,dN=N(\mu -\gamma )+c$$

where c is integration constant, *γ* has dimension of energy. Integration constant is conditionally set as zero because it is related to reference point.

*γ* is obtained from energy of ionization (I) and electron affinity (A)

11 $${\rm{I}}={\rm{E}}(N-1)-{\rm{E}}(N),\,{\rm{A}}={\rm{E}}(N)-{\rm{E}}(N+1)$$

and Eq. (9) as follows

12 $${\rm{\gamma }}=\frac{I-A}{(N-1)ln\frac{N-1}{N}+(N+1)ln\frac{N+1}{N}}$$

13 $${\mu }_{0}=-I+\gamma (N-1)ln\frac{N-1}{N}$$

### Model of total energy calculation of a molecule

For a start, according to the PCP concept, the electrons are localized into atoms (domain) so that the molecules become the set of atoms with different number of electrons, in order to apply the thermodynamic formalization method by making molecules multi-phase one-component system where the localized atoms are deemed as phase and the electrons as component in temperature zero limit thermal equilibrium system.

In Born-Oppenheimer approximation, the total energy of molecules Hamiltonian is given as follows:

14 $$\hat{H}={\hat{T}}_{e}+{\hat{V}}_{ne}+{\hat{V}}_{ee}+{\hat{V}}_{nn}$$

To construct multi-phase one-component system model, the electron coordinates are replaced by nucleus ones by setting distance between electrons as ${r}_{\alpha i}-{r}_{\beta j}\approx {R}_{\alpha \beta }$ in integral Equations. As a result, the total space integral values for the squared wave function are electron number N, where ${\overrightarrow{r}}_{\alpha i}-{\overrightarrow{r}}_{\beta j}$ is the distance between *i*-th electron of *α*-th atom and *j*-th electron of *β*-th atom, and ${\overrightarrow{R}}_{\alpha \beta }$ is the distance between *α*-th atom and *β*-th atom. To figure out the average value of energy;

Kinetic energy of electrons in molecule is the sum of kinetic energy of electrons in atom.

15 $${T}_{e}=\sum _{\alpha }{t}_{e,\alpha }$$

The nucleus-electron interaction energy in molecule V_*ne*_ is the sum of interaction terms of V_*ne*,*a*_ in domains or those of domains.

16 $$\begin{array}{c}{V}_{ne}\approx \sum _{\alpha }{V}_{ne,\alpha }-\sum _{\alpha }\sum _{\beta }\frac{{Z}_{\alpha }{N}_{\beta }}{{R}_{\alpha \beta }}\end{array}$$

where *Z*_α_ is charge of α atom, *N*_*β*_ is electron number of *β* atom domain, *V*_*ne*,*a*_ is nucleus-electron interaction energy in α atom domain.

If density matrix is approximated to *ρ* (*r*_1_, *r*_2_) ≈ *ρ* (*r*_1_) *ρ* (*r*_2_), electron-electron interaction energy is also the sum of two term.

17 $$\begin{array}{c}{V}_{ee}\approx \sum _{\alpha }{v}_{ee,\alpha }+\frac{1}{2}\sum _{\alpha }\sum _{\beta \ne \alpha }\frac{{N}_{\alpha }{N}_{\beta }}{{R}_{\alpha \beta }}\end{array}$$

The nucleus-nucleus interaction term is

18 $${V}_{nn}=\frac{1}{2}\sum _{\alpha }\sum _{\beta \ne \alpha }\frac{{Z}_{\alpha }{Z}_{\beta }}{{R}_{\alpha \beta }}$$

Therefore, from Eqs. 15~18, total energy allotted to atom domain is represented as follows.

19 $$\begin{array}{c}E\approx \sum _{\alpha }({t}_{e,\alpha }+{v}_{ee,\alpha }+{v}_{ne,\alpha })+\\ \,{\boldsymbol{+}}\frac{1}{2}\sum _{\alpha }(-2{Z}_{\alpha }\sum _{\beta \ne \alpha }\frac{{N}_{\beta }}{{R}_{\alpha \beta }}+{N}_{\alpha }\sum _{\beta \ne \alpha }\frac{{N}_{\beta }}{{R}_{\alpha \beta }}+{Z}_{\alpha }\sum _{\beta \ne \alpha }\frac{{Z}_{\beta }}{{R}_{\alpha \beta }})\end{array}$$

If introducing atom charge ${q}_{\alpha }={Z}_{\alpha }-{N}_{\alpha }$ and taking into account $\sum _{\alpha }\sum _{\beta \ne \alpha }{N}_{\alpha }{Z}_{\beta }\,=\,\sum _{\alpha }\sum _{\beta \ne \alpha }{N}_{\beta }{Z}_{\alpha }$, the total energy of molecule *E* is given as follows, where the sum of the total energy in α atom domain is represented by the sum of interaction term between *E*_*α*_ and atom domains;

20 $$E\approx \sum _{\alpha }{E}_{\alpha }+\frac{1}{2}\sum _{\alpha }\sum _{\beta \ne \alpha }\frac{{q}_{\alpha }{q}_{\beta }}{{R}_{\alpha \beta }}$$

### Equation to calculate molecular energy by means of PCP

Total energy of molecule is approximately calculated, but it is based on quantum chemical average value of total energy operator. Therefore, total energy of molecule is represented by sum of energy per atom domain and electrostatic interaction energy between domains.

By substituting Eq. (10) for Eq. (20), total energy of molecule, following Eq. is obtained.

21 $${E}^{M}\approx \sum _{\alpha }[{N}_{\alpha }({\mu }_{\alpha }^{0}+{\gamma }_{\alpha }\,{ln}\,\frac{{N}_{\alpha }}{{N}_{\alpha }^{0}}-{\gamma }_{\alpha })+\frac{1}{2}\sum _{\beta \ne \alpha }\frac{{q}_{\alpha }{q}_{\beta }}{{R}_{\alpha \beta }}]$$

where *E*^*M*^ has no property to calculate the quantum chemical average value of operator, but the thermodynamic formulization only. In addition, *E*^*M*^ cannot be assumed to be the total energy of molecule like Eq. (20). Because PCP is energy to each electron numerically, it is not an absolute value of energy but a variation proportion. *E*^*M*^ in Eq. (21) is defined as molecular energy and used as initial equation. Regarding a pure-state molecule as a multi-phase one-component system demands complicate approximation, which means it would not be suitable for quantitative calculation. To obtain a correct value, correction factor (parameter) *k*_*αβ*_ is applied.

22 $${E}^{M}=\sum _{\alpha }[{N}_{\alpha }({\mu }_{\alpha }^{0}+{\gamma }_{\alpha }\,{ln}\,\frac{{N}_{\alpha }}{{N}_{\alpha }^{0}}-{\gamma }_{\alpha })]+\sum _{\alpha }\sum _{\beta \ne \alpha }{k}_{\alpha \beta }\frac{{q}_{\alpha }{q}_{\beta }}{{R}_{\alpha \beta }}$$

where *k*_*αβ*_ is an interaction parameter between α atom and β atom.

Parameter *k*_*αβ*_ of Eq. (22) is successfully estimated, and database is constructed.

### Pseudo chemical potential of each phase (atom) (PCPP) in molecule

First, according to the common method in thermodynamics, pseudo chemical potential of each (atom) phase (PCPP) ${\mu }_{\alpha }^{M}$ that composes molecules is determined by calculating partial derivative to Eq. 23.

23 $${(\frac{\partial {E}^{M}}{\partial {N}_{\alpha }})}_{V,{N}_{\beta \ne \alpha }}={\mu }_{\alpha }^{M},\,{\mu }_{\alpha }^{M}={\mu }_{\alpha }^{0}+{\gamma }_{\alpha }\,\mathrm{ln}\,\frac{{N}_{\alpha }}{{N}_{\alpha }^{0}}-\sum _{\beta }{k}_{\alpha \beta }\frac{{q}_{\beta }}{{R}_{\alpha \beta }}$$

where ${\mu }_{\alpha }^{M}$ is the PCPP of α-th phase and the function of N and *R*_*αβ*_ as well.

How to form a molecule from atoms is interpreted by variable principle to variable quantity *N*.

$${{\rm{E}}}_{0}={\rm{E}}({N}_{0},v(r))\le E(N,v(r))$$

The formation process of the molecules from atoms is a minimizing process of energy by migration of electrons between the phases (atoms), that is the equalizing process of ${\mu }_{\alpha }^{M}$. Because state of minimum energy is a temperature zero limit thermoequilibrium state, in the closed system,

24 $${\rm{d}}{E}^{M}={\sum }_{\alpha =1}^{p}{\mu }_{\alpha }^{M}d{N}_{\alpha }={\mu }_{1}^{M}d{N}_{1}+{\mu }_{2}^{M}d{N}_{2}+\ldots +{\mu }_{p}^{M}d{N}_{p}=0$$

25 $$\begin{array}{rcl}{\rm{N}} & = & {\sum }_{\alpha =1}^{p}{N}_{\alpha }={N}_{1}+{N}_{2}+{N}_{2}+\ldots +{N}_{p}={\rm{c}}\\ {\rm{dN}} & = & {\rm{d}}({\sum }_{\alpha =1}^{p}{N}_{\alpha })=d{N}_{1}+d{N}_{2}+d{N}_{2}+\ldots +d{N}_{p}=0,\end{array}$$

(p: number of phases (atoms), α: kind of phase (atom), *N*_*α*_: electron number of α-th phase (atom), N: electron number of molecule)

26 $$((1)-(2)\times {\mu }_{1}^{M})=({\mu }_{2}^{M}-{\mu }_{1}^{M})d{N}_{2}+({\mu }_{2}^{M}-{\mu }_{1}^{M})d{N}_{3}+\ldots ({\mu }_{p}^{M}-{\mu }_{1}^{M})d{N}_{p}=0,$$

from $$d{N}_{\alpha }\ne 0,$$ relationship

27 $${\mu }_{1}^{M}={\mu }_{2}^{M}=\ldots ={\mu }_{\alpha }^{M}=\ldots ={\mu }_{p}^{M}$$

could be obtained.

According to the principle that charge or number of particles are also conserved in molecules as in the closed thermodynamic system, the following equation must be given;

28 $$\mathop{\sum }\limits_{\alpha =1}^{n}{q}_{\alpha }=0\,{\rm{or}}\,\mathop{\sum }\limits_{\alpha =1}^{n}{N}_{\alpha }=N$$

The simultaneous Eq. (29) is defined as charge distribution equation, where the number of electrons is an undefined *N*_*α*_(23, 27, 28).

29 $$\{\begin{array}{c}{\mu }_{1}^{0}+{\gamma }_{1}\,\mathrm{ln}\,\frac{{N}_{1}}{{N}_{1}^{0}}-\sum _{\alpha }{k}_{1\beta }\frac{{q}_{\beta }}{{R}_{1\beta }}={\mu }^{M}\\ {\mu }_{2}^{0}+{\gamma }_{2}\,\mathrm{ln}\,\frac{{N}_{2}}{{N}_{2}^{0}}-\sum _{\beta }{k}_{2\beta }\frac{{q}_{\beta }}{{R}_{2\beta }}={\mu }^{M}\\ {\mu }_{n}^{0}+{\gamma }_{n}\,\mathrm{ln}\,\frac{{N}_{n}}{{N}_{n}^{0}}-\sum _{\beta }{k}_{n\beta }\frac{{q}_{\beta }}{{R}_{n\beta }}={\mu }^{M}\\ \mathop{\sum }\limits_{\alpha =1}^{n}{q}_{\alpha }=0\end{array}$$

Atom charge *q*_α_ and *N*_*α*_ in molecule are calculated and molecule energy is determined by solving Eq. (29) by Newton-Raphson method. The number of electrons, or the solution of simultaneous equation, is the stationary value that gives the minimum value of energy and also the one that determines atom charge in ground state.

## Results and Discussion

### Efficiency of PCP method

Total energy of some molecules calculated by PCP method and their calculation duration is shown in Table 1. Comparing against other methods, this method is fast in calculation speed and high in accuracy. The calculation duration gets longer when the atoms in the molecules are big in number. For instance, in case of the ab initio method and DFT method, when each of them is given more than 130 and 30 atoms respectively, it is infeasible due to their prolonged calculation duration. Furthermore, the results of this method are very similar to the one of ab initio method and DFT method, indicating that it is higher in accuracy.

**Table 1:** The total energies and calculation speeds of some molecules obtained by using different calculating methods.

Moleclues	The Number of atoms	Total Energy/eV (Calculation Time^†^)									
		Our method		ab initio				DFT			
			time	STO-3G	time	6–31 G**	time	STO-3G	time	6–31 G**	time
C_2_H_5_OH	9	−4 163.42	1	−4 138.03.	2	−4 191.28	6	−4 159.84	11	−4 214.66	74
C_5_H_11_ON	18	−8 759.92	5	−8 730.63	2	−8 841.32	62	−8 778.17	98	−8 891.79	764
C_8_H_16_O_2_N_2_	28	−15 363.27	9	−15 330.33	8	−15 525.15	3456	−15 411.63	401	—	—
C_18_H_31_O_7_N_7_	63	−42 984.28	17	−43 084.45	79	−43 136.85	7200	—	—	—	—
C_38_H_67_O_13_N_11_	129	−81 267.30	80	−82 946.57	5441	—	—	—	—	—	—
C_59_H_106_O_18_N_19_	202	−123 532.11	195	—	—	—	—	—	—	—	—
C_89_H_160_O_28_N_26_	303	−182 461.09	365	—	—	—	—	—	—	—	—

^†^Calculation time is the relative value.

### Evaluation of accuracy

To further evaluate its accuracy, it is compared to the ab initio method, calculating the total energies of some organic and inorganic molecules, as shown in Table 2 and Table 3, respectively. The results in their Tables clearly show that calculation results of the new method are approximate to one of ab initio methods, and on the other hand, the relative errors in calculating value of all organic and inorganic molecules are rare (lower than 0.0176), indicating that PCP method is accurate in calculation of total energy of the molecules.

**Table 2:** Calculation results of total energy of some organic molecules.

Molecules	Our method/eV	Ab initio^#^/eV	Absolute error/eV	Relative error
i-C_4_H_9_OH	−6 254.015 0	−6 239.385 0	−14.630 0	0.002 3
C_2_H_5_CHO	−5 182.359 0	−5 157.137 3	−25.221 7	0.004 9
C_3_H_7_NHC_2_H_5_	−6 750.746 2	−6 758.033 6	7.287 4	−0.001 1
a-C_6_H_5_C_2_H_4_OH	−10 328.641 3	−10 309.678 1	−18.963 1	0.001 8
i-C_4_H_9_COOH	−9 304.506 3	−9 266.696 1	−37.810 3	0.004 1
C_5_H_12_	−5 267.672 4	−5 280.168 1	12.495 7	−0.002 4
C_2_H_5_C≡CH	−4 174.285 7	−4 164.064 4	−10.221 3	0.002 4
C_2_H_5_CN	−4 614.964 1	−4 594.626 6	−20.337 4	0.004 4
c-C_6_H_11_CH_3_	−7 326.771 5	−7 348.687 2	21.915 7	−0.003 0
C_5_H_11_COOC_2_H_5_	−12 433.413 4	−12 416.240 0	−17.173 4	0.001 4
C_3_H_7_OC_2_H_5_	−7 294.404 3	−7 289.192 5	−5.211 8	0.000 7
CF_4_	−11 863.122 9	−11 689.321 1	−173.801 9	0.014 7
CH_3_COC_4_H_9_	−8 321.452 6	−8 306.837 4	−14.615 2	0.001 8
C_4_H_9_NO_2_	−9 738.860 1	−9 691.735 9	−47.124 2	0.004 8
CH_2_=CHCH_2_SH	−13 933.442 3	−13 845.659 8	−87.782 5	0.006 3

^#^STO-3G.

**Table 3:** Calculation results of total energy of some inorganic molecules.

Molecules	Our method/eV	Ab initio^#^/eV	Absolute error/eV	Relative error
H_2_CO_3_	−7 155.095 2	−7 076.963 1	−78.132 0	0.011 0
H_2_CrO_4_	−36 446.291 2	−36 163.705 6	−282.585 5	0.007 8
H_2_O	−2 078.832 3	−2 039.895 7	−38.936 5	0.019 1
H_2_SO_3_	−16 909.145 7	−16 753.253 0	−155.892 7	0.009 3
H_2_SO_4_	−18 948.070 2	−18 757.919 4	−190.150 8	0.010 1
H_3_AsO_3_	−66 805.531 9	−66 185.507 3	−620.024 6	0.009 4
H_3_AsO_4_	−68 782.011 9	−68 198.005 1	−584.006 8	0.008 6
H_3_BO_3_	−6 820.143 2	−6 742.656 9	−77.486 3	0.011 5
H_3_PO_4_	−17 519.650 5	−17 248.984 0	−270.666 5	0.015 7
H_4_P_2_O_7_	−33 028.464 4	−32 458.767 0	−569.697 5	0.017 6
HClO_2_	−16 561.938 5	−16 342.648 8	−219.289 7	0.013 4
HClO	−14 524.665 9	−14 392.737 5	−131.928 4	0.009 2
HMnO_4_	−39 347.431 5	−39 005.056 8	−342.374 7	0.008 8
HNO_2_	−5 562.993 6	−5 494.318 5	−68.675 1	0.012 5
Ca(NO_3_)_2_	−33 597.275 9	−33 206.266 1	−391.009 8	0.011 8
Fe(NO_3_)_2_	−49 507.850 7	−48 953.044 7	−554.806 0	0.011 3
FeCl_2_	−59 291.348 3	−58 719.101 3	−572.247 0	0.009 7
FeSO_4_	−53 244.442 0	−52 705.067 5	−539.374 6	0.010 2
KMnO_4_	−55 600.162 8	−55 128.824 3	−471.338 5	0.008 5
Mg(NO_3_)_2_	−20 671.974 8	−20 343.118 1	−328.856 7	0.016 2
MgSO_4_	−24 402.032 0	−24 092.248 5	−309.783 5	0.012 9
Na_2_SO_4_	−27 615.986 5	−27 433.242 0	−182.744 5	0.006 7
NaNO_3_	−11 914.849 1	−11 838.296 2	−76.552 9	0.006 5
ZnSO_4_	−67 241.239 2	−66 545.293 8	−695.945 4	0.010 5

**Figure 1:**
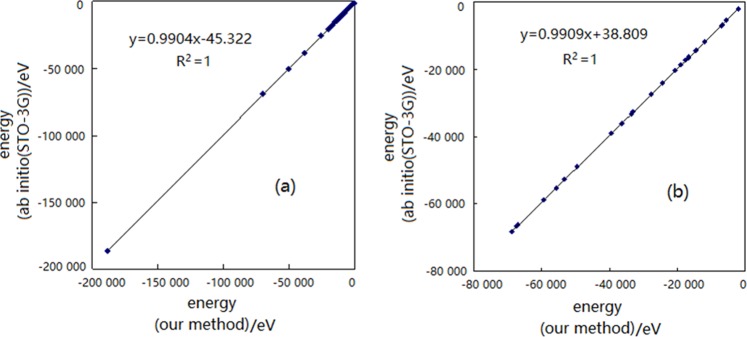
The result of correlation analysis for total energies of some molecules. (**a**) organic molecules. (**b**) inorganic molecules.

Figure 1 shows the correlation analysis of the total energies of some molecules, organic (Fig. 1a) and inorganic (Fig. 1b). Regression equations are expressed as follows, respectively:

30 $$y=0.9904x-45.322$$

31 $$y=0.9909x-38.809$$

Their correlation coefficients R^2^ are all 1 and the linearities are very good in both cases. These results also prove the accuracy of the new calculation method.

Tables 4 and 5 show comparison calculations of atomic charge distributions of some organic and inorganic molecules using 3 different methods. The results of Tables 4 and 5 are approximate to the results above, revealing that for calculation of the atomic charge distribution, the result of the new method is similar to one of the ab initio.

**Table 4:** Comparison of calculation results for atomic charge distribution of some organic molecules.

Molecule	Atom	Charge		
		ab initio^#^	Our method	PM3
C_3_H_7_OH	C^1^	−0.178 8	−0.158 2	−0.106 5
	C^2^	−0.099 3	−0.088 9	−0.113 8
	C^3^	0.007 7	0.017 5	0.073 6
	O^4^	−0.288 4	−0.325 3	−0.311 6
	H^5^	0.061 4	0.051 9	0.039 5
	H^6^	0.057 5	0.052 3	0.036 9
	H^7^	0.057 5	0.052 3	0.036 9
	H^8^	0.061 2	0.062 3	0.060 1
	H^9^	0.061 2	0.062 3	0.060 1
	H^10^	0.044 6	0.052 3	0.020 3
	H^11^	0.044 6	0.052 3	0.020 3
	H^12^	0.170 5	0.169 2	0.184 1
	R^2^		0.988 9	0.926 9
C_2_H_6_	C^1^	−0.174 8	−0.161 3	−0.105 6
	H^2^	0.058 3	0.053 8	0.035 2
	C^s^	−0.174 8	−0.161 3	−0.105 6
	H^4^	0.058 3	0.053 8	0.035 2
	H^5^	0.058 3	0.053 8	0.035 2
	H^6^	0.058 3	0.053 8	0.035 2
	H^7^	0.058 3	0.053 8	0.035 2
	H^8^	0.058 3	0.053 8	0.035 2
	R^2^		1.000 0	1.000 0
C_2_H_5_NH_2_	C^1^	−0.178 8	−0.168 5	−0.111 4
	C^2^	−0.015 5	−0.083 7	−0.098 0
	N^3^	−0.379 9	−0.285 7	−0.037 6
	H^4^	0.060 0	0.044 9	0.038 7
	H^5^	0.069 4	0.061 7	0.050 0
	H^6^	0.056 4	0.058 0	0.036 3
	H^7^	0.060 5	0.072 9	0.053 0
	H^8^	0.041 5	0.068 3	0.023 5
	H^9^	0.143 8	0.113 7	0.023 0
	H^10^	0.142 6	0.118 3	0.022 5
	R^2^		0.950 4	0.378 8
**Molecule**	**Atom**	**Charge**		
		**ab initio**^**#**^	**Our method**	**PM3**
i−C_4_H_9_CHO	C^1^	−0.014 1	−0.031 3	−0.065 9
	C^2^	−0.129 1	−0.131 2	−0.185 6
	C^3^	0.138 2	0.132 5	0.271 3
	O^4^	−0.211 5	−0.292 0	−0.316 7
	H^5^	0.062 4	0.059 9	0.076 1
	H^6^	0.066 8	0.071 5	0.081 1
	H^7^	0.045 0	0.056 6	0.053 7
	H^8^	0.054 1	0.061 7	0.063 5
	C^9^	−0.182 1	−0.136 8	−0.116 7
	H^10^	0.059 5	0.056 7	0.043 8
	H^11^	0.060 9	0.062 3	0.044 6
	H^12^	0.057 6	0.066 5	0.040 7
	C^13^	−0.182 5	−0.149 8	−0.122 8
	H^14^	0.060 5	0.056 9	0.047 2
	H^15^	0.056 7	0.059 6	0.043 7
	H^16^	0.057 6	0.056 8	0.041 9
	R^2^		0.949 0	0.847 6
C_2_H_3_CH_3_	C^1^	−0.141 2	−0.149 4	−0.172 1
	H^2^	0.060 2	0.060 1	0.079 6
	C^3^	−0.047 1	−0.042 8	−0.134 4
	H^4^	0.057 7	0.064 2	0.080 6
	C^5^	−0.186 4	−0.170 5	−0.078 1
	H^6^	0.058 1	0.058 8	0.094 5
	H^7^	0.063 8	0.057 3	0.038 9
	H^8^	0.067 4	0.061 2	0.045 4
	H^9^	0.067 4	0.061 2	0.045 4
	R^2^		0.994 7	0.723 7
CH_2_Cl_2_	C^1^	−0.027 9	−0.023 1	−0.106 1
	Cl^2^	−0.117 8	−0.121 2	−0.021 7
	Cl^3^	−0.117 8	−0.121 2	−0.021 7
	H^4^	0.131 8	0.132 8	0.074 7
	H^5^	0.131 8	0.132 8	0.074 7
	R^2^		0.999 5	0.521 5

^#^STO−3G.

**Table 5:** Comparison of calculation results for atomic charge distribution of some inorganic molecules.

molecule	atom	charge		
		ab initio^#^	PCP	PM3
H_2_CO_3_	C^1^	0.420 3	0.307 3	0.492 2
	O^2^	−0.279 7	−0.249 8	−0.244 0
	O^3^	−0.278 7	−0.200 1	−0.387 7
	O^4^	−0.304 2	−0.265 5	−0.327 4
	H^5^	0.221 1	0.202 8	0.224 8
	H^6^	0.221 2	0.205 2	0.242 1
H_2_SO_4_	S^1^	0.810 4	1.219 0	2.508 3
	O^2^	−0.304 6	−0.434 7	−0.656 3
	O^3^	−0.322 4	−0.410 9	−0.862 5
	O^4^	−0.304 5	−0.434 4	−0.656 3
	H^5^	0.221 2	0.235 9	0.264 7
	O^6^	−0.321 5	−0.410 8	−0.862 5
	H^7^	0.221 3	0.235 9	0.264 6
H_4_P_2_O_7_	P^1^	1.255 2	1.182 3	2.271 8
	O^2^	−0.561 8	−0.475 8	−1.035 5
	P^3^	1.255 7	1.183 4	2.271 9
	O^4^	−0.524 7	−0.477 8	−0.901 3
	O^5^	−0.524 5	−0.477 3	−0.901 3
	O^6^	−0.419 4	−0.428 0	−0.657 1
	O^7^	−0.419 5	−0.428 1	−0.657 1
	O^8^	−0.419 5	−0.428 0	−0.657 1
	O^9^	−0.419 3	−0.427 9	−0.657 0
	H^10^	0.194 4	0.194 4	0.230 7
	H^11^	0.194 5	0.194 3	0.230 7
	H^12^	0.194 5	0.194 3	0.230 7
	H^13^	0.194 4	0.194 3	0.230 6
KOH	K^1^	0.402 0	0.369 6	—
	O^2^	−0.423 5	−0.395 4	—
	H^3^	0.021 4	0.025 8	—
ZnSO_4_	S^1^	0.865 2	0.647 9	2.460 6
	O^2^	−0.498 5	−0.480 4	−0.727 9
	O^3^	−0.454 9	−0.480 5	−0.728 0
	O^4^	−0.338 6	−0.247 3	−0.853 6
	O^5^	−0.338 7	−0.247 3	−0.853 6
	Zn^6^	0.765 4	0.807 5	0.702 5
H_3_AsO_4_	O^1^	−0.426 7	−0.456 2	−0.690 8
	O^2^	−0.426 8	−0.456 4	−0.691 6
	As^3^	1.245 5	1.397 2	2.086 9
	O^4^	−0.510 0	−0.555 3	−0.775 0
	O^5^	−0.426 9	−0.456 4	−0.690 4
	H^6^	0.181 7	0.175 6	0.253 8
	H^7^	0.181 5	0.175 7	0.253 6
	H^8^	0.181 7	0.175 7	0.253 6
H_2_CrO_4_	Cr^1^	1.326 4	1.326 4	−0.698 2
	O^2^	−0.398 3	−0.396 0	0.141 3
	O^3^	−0.409 1	−0.411 5	0.126 1
	O^4^	−0.469 3	−0.469 3	−0.019 2
	O^5^	−0.469 3	−0.469 3	−0.019 3
	H^6^	0.209 7	0.209 8	0.234 7
	H^7^	0.209 8	0.209 8	0.234 7
Be(OH)_2_	Be^1^	0.404 3	0.406 5	0.614 2
	O^2^	−0.416 3	−0.401 4	−0.499 9
	O^3^	−0.416 3	−0.401 4	−0.499 9
	H^4^	0.214 2	0.198 2	0.192 8
	H^5^	0.214 2	0.198 1	0.192 8
Ca(OH)_2_	Ca^1^	0.668 8	0.743 4	—
	O^2^	−0.410 4	−0.440 8	—
	O^3^	−0.410 4	−0.440 8	—
	H^4^	0.076 0	0.069 1	—
	H^5^	0.076 0	0.069 1	—
Mg(NO_3_)_2_	N^1n^	0.201 1	0.330 7	1.379 0
	O^n^	−0.104 6	−0.212 6	−0.530 3
	O^n^	−0.312 6	−0.309 0	−0.641 2
	O^n^	−0.312 5	−0.360 0	−0.640 9
	Mg^n^	1.057 3	1.102 1	0.866 9
	N^n^	0.201 1	0.330 7	1.379 0
	O^7^	−0.104 6	−0.212 6	−0.530 3
	O^8^	−0.312 6	−0.309 0	−0.641 2
	O^9^	−0.312 5	−0.360 0	−0.640 9
R^2^			0.964	0.846

^#^STO-3G.

## Conclusions

In summary, we have proposed a novel method for calculation of the molecular electron system by thermodynamic formalization rather than quantum chemical method.

The molecular model of multiple-phase one-component system has been proposed which takes atoms as phase and electrons as component in the molecule. A model of molecular energy has been proposed based on a new concept of pseudo chemical potential (PCP) and pseudo chemical potential of component (electron) of each phase (atom) (PCPP) in multi-phase one-component have been defined. The simultaneous equation obtained from equilibrium condition of PCPP is just the variational equation. The validity of the thermodynamic formalization is verified through calculation experiment.

The calculation results show that the new idea is valid and very efficient for calculating the molecular systems. It has potentials to be further developed and applied in some fields, such as some physicochemical properties, chemical reactions, and catalyst, etc.

## Methods

### PCP method

#### Parameter Estimation

Parameters of neutral atoms ${\mu }_{0},\gamma$

They are experimentally determined by using electron affinity and ionization energy.

#### Interaction parameters

They are determined by least squares so that the atomic charges and energies of some sample molecules, using equation of atomic charge calculation, could reproduce result of calculation by ab initio (STO-3G).

### Calculation method of the atomic distribution and total energy of a molecule

Since the molecular charge calculation Eq. (29) is a nonlinear spline equation, it can be solved by using the typical Newton-Raphson method.

For this purpose, Eq. (29) is rewritten into the following form.

32 $$\{\begin{array}{c}{\mu }_{1}({\bf{N}})=0\\ {\mu }_{2}({\bf{N}})=0\\ \cdots \cdots \cdots \\ {\mu }_{n}({\bf{N}})=0\\ {\mu }_{{\rm{n}}+1}({\bf{N}})=0\end{array}$$

where $${\bf{N}}=({N}_{1},{N}_{2},\cdots ,{N}_{n},{\mu }^{M})$$

33 $$\{\begin{array}{c}{\mu }_{i}({\bf{N}})=-{\mu }^{M}+{\mu }_{i}^{0}+{\gamma }_{i}ln\frac{{N}_{i}}{{N}_{i}^{0}}-\sum _{j\ne i}{k}_{ij}\frac{{q}_{j}}{{R}_{ij}}(i=\overline{1,n})\\ {\mu }_{n+1}({\bf{N}})=\mathop{\sum }\limits_{i=1}^{n}{q}_{i}\end{array}$$

However, when the molecular charge calculation Eq. (33) is repeated because of the logarithms in the equation, if the values are negative in the iteration process, they would not converge and thus the calculation would go wrong. In order to overcome this problem, Eq. (33) is rewritten as follows.

First, let ${ln}\,\frac{{N}_{i}}{{N}_{i}^{0}}=N{a}_{i}$, then the following Eq. (34) is given;

34 $${N}_{i}={N}_{i}^{0}\exp (N{a}_{i}),\,{q}_{i}={N}_{i}^{0}-{N}_{i}={N}_{i}^{0}[1-\exp (N{a}_{i})]$$

Thus, Eq. (34) can be rewritten as follows;

35 $$\{\begin{array}{c}{\mu }_{1}({\boldsymbol{Na}})=0\\ {\mu }_{2}({\boldsymbol{Na}})=0\\ \cdots \cdots \cdots \cdots \\ {\mu }_{n}({\boldsymbol{Na}})=0\\ {\mu }_{n+1}({\boldsymbol{Na}})=0\end{array}$$

36 $$\{\begin{array}{c}{\mu }_{i}({\boldsymbol{Na}})=-{\mu }^{M}+{\mu }_{i}^{0}+{\gamma }_{i}N{a}_{i}-\sum _{j}{k}_{ij}\frac{{N}_{j}^{0}\,[1-\exp (N{a}_{j})]}{{R}_{ij}}\\ {\mu }_{n+1}({\boldsymbol{Na}})=\mathop{\sum }\limits_{i=1}^{n}{N}_{i}^{0}[1-{\exp }(N{a}_{i})]\end{array}(i=\overline{1,n})$$

Based on the spline equation above, the algorithm for calculating the atomic charge was constructed as follows;

Read the value of the neutral atom parameter ${\mu }_{i}^{0},\,{\gamma }_{i}$ and the number of electrons in the neutral atom ${N}_{i}^{0}$, and set the initial value $N{{a}_{i}}^{(k-1)},{\mu }^{M(k-1)}$.

$$\begin{array}{c}N{a}_{i}^{(k-1)}{\boldsymbol{=}}0\,(i=\overline{1,n})\\ {\mu }^{M(k-1)}=\frac{1}{n}\mathop{\sum }\limits_{i=1}^{{\rm{n}}}{\mu }_{i}^{0}\end{array}$$

And then, solve the following equation by using solve method of MATLAB function library;

37 $$\{\begin{array}{c}{\mu }_{i}({\boldsymbol{N}})=-{\mu }^{M}+{\mu }_{i}^{0}+{\gamma }_{i}ln\frac{{N}_{i}}{{N}_{i}^{0}}-\sum _{j\ne i}{k}_{ij}\frac{{q}_{j}}{{R}_{ij}}\,(i=\overline{1,n})\\ {\mu }_{n+1}({\boldsymbol{N}})=\mathop{\sum }\limits_{i=1}^{n}{q}_{i}\end{array}$$

Thereafter, the function values of each atom can be calculated;

38 $${{\mu }_{i}}^{(k-1)}={{\mu }_{i}({\boldsymbol{Na}})|}_{{\boldsymbol{Na}}={\boldsymbol{N}}{{\boldsymbol{a}}}^{(k-1)}},\,(i=\overline{1,{\rm{n}}{\boldsymbol{+}}1})$$

At this time, the inter-nucleus distances are calculated from molecular structure data obtained by molecular-dynamics method as follows;

39 $${R}_{ij}=\sqrt{{({x}_{i}-{x}_{j})}^{2}+{({y}_{i}-{y}_{j})}^{2}+{({z}_{i}-{z}_{j})}^{2}}$$

Atomic charge ${q}_{i}(i=\overline{1,n})$ is calculated from ${N}_{i}={N}_{i}^{0}\exp (N{a}_{i})$ and ${q}_{i}={N}_{i}^{0}-{N}_{i}={N}_{i}^{0}[1-{\exp }(N{a}_{i})]$

On the other hand, according to the atomic charge calculated by using the method above, total energy of the molecule can be calculated as follows;

40 $${E}^{M}=\sum _{\alpha }[{N}_{\alpha }({\mu }_{\alpha }^{0}+{\gamma }_{\alpha }\,{ln}\,\frac{{N}_{\alpha }}{{N}_{\alpha }^{0}}-{\gamma }_{\alpha })]-\sum _{\alpha }\sum _{\beta \ne \alpha }{k}_{\alpha \beta }\frac{{q}_{\alpha }{q}_{\beta }}{{R}_{\alpha \beta }}$$

In addition, PCPs of the molecules are also calculated in the calculation process.

### Quantum chemical methods

Quantum chemical calculations have been carried out by using the ab initio (STO-3G, 6–31 G**), DFT (STO-3G, 6–31 G**) in “Gaussian 09 W” and semi-empirical PM3 in “Hyperchem 8.0.3”.
